# Value of anti-p53 antibody as a biomarker for hepatocellular carcinoma: Evidence from a meta-analysis

**DOI:** 10.1097/MD.0000000000021887

**Published:** 2020-08-21

**Authors:** Yue Chang, Baiqing Liu, Haiyan Niu, Zhenguo Wang, Shihai Xia, Hai Li

**Affiliations:** a Department of Hepatopancreatobiliary and Splenic Medicine, Characteristic Medical Center of People's Armed Police Force; b Tianjin Key Laboratory of Hepatopancreatic Fibrosis and Molecular Diagnosis and Treatment; c Division of Gastroenterology and Hepatology, Tianjin Xiqing Hospital, Tianjin, China.

**Keywords:** biomarker, diagnosis, hepatocellular carcinoma, meta-analysis, p53 antibody

## Abstract

**Introduction:**

The incidence of hepatocellular carcinoma (HCC) ranks sixth in the world, but its mortality is the third highest due to the lack of early diagnostic markers. Nowadays, the increase of autoantibody levels has been found in many cancers, and many studies have begun to pay attention to the detection of anti-p53 antibodies in HCC. The purpose of this study is to quantitatively and comprehensively analyze the potential diagnostic value of anti-p53 autoantibodies in HCC

**Methods:**

English articles up to November 2019 were collected. The overall sensitivity and specificity were calculated. Besides, the positive likelihood ratio, negative likelihood ratio (NLR), diagnostic odds ratio (DOR), and summary receiver operating characteristic curves of the overall diagnostic accuracy of anti-p53 antibody were calculated by STATA software. Finally, according to the heterogeneity of the results, the subgroup analysis, and the publication bias were performed.

**Results:**

A total of 16 eligible studies were incorporated into this meta-analysis, including 1323 patients with HCC and 1896 control. The pooled sensitivity was 0.28(0.17–0.41) and specificity was 0.98 (0.95–0.99). The pooled DOR was 10.44 (6.31–17.29) and the pooled NLR was 0.74 (0.63–0.86). The area under ROC curve of symmetrical ROC was 0.840.

**Conclusions:**

The anti-p53 antibody has a high specificity for HCC, but the low sensitivity is not perfect and would limit the clinical application. The anti-p53 antibody would help rule out HCC but not help rule in HCC for early diagnosis. Whether combined as a diagnostic panel with other biomarkers or laboratory tests may prove useful requires further study.

## Abstract 2

Supplemental Digital Content is available in the text

## 1 Introduction

Hepatocellular carcinoma (HCC) is the sixth most common malignant tumor in the world, and most patients will die in 1 year after the clear diagnosis of HCC.^[1]^ The third high mortality rate of HCC is partly due to the lack of effective early diagnosis technology for HCC around the world. There is a high incidence of HCC, about 50.5% of new patients and 51.4% HCC-related deaths globally occur in China every year.^[2]^ In current clinical practice, alpha-fetoprotein (AFP) and imaging characteristics are the most commonly used modality for diagnosis and monitoring of HCC.^[3,4]^ However, AFP cannot be effective in differentiating HCC and other liver diseases, suggesting that the effect of screening is generally in the early diagnosis of HCC.^[5]^ As many as 40% of patients with normal AFP levels cannot be accurately detected in early stage and depends on definitive imaging results. This often leads to misdiagnosis or a delay in the correct diagnosis and management.^[6]^ Therefore, it is urgent to find a novel noninvasive or less invasive biomarker, known as “liquid biopsy,” to detect HCC at an early stage.^[7]^

An increase in serum autoantibody levels has been shown to precede the occurrence and development of cancers. Immunologic processes causing autoantibody production are believed to be generated by the immune system in response to mutations, overexpression of proteins, degradation, or others.^[8,9]^ Therefore, there is a growing interest in autoantibody levels in patient blood serum as noninvasive diagnostic biomarkers for early-stage diagnosis of HCC.^[10]^ P53 protein is encoded by the tumor suppressor P53 gene, which plays an important role in cell cycle regulation, cell apoptosis, DNA repair, and angiogenesis and has been studied in various types of cancer for many years. However, p53 gene mutations caused by various reasons can lead to the accumulation of abnormal p53 protein,^[11]^ which act as antigens that may produce different levels of anti-p53 antibodies in serum, tissue, cell, and other body fluids.^[12]^ According to the report, the proportion of the p53 mutations and anti-p53 antibody positive is more than 50% in patients with HCC.^[4,13]^

Along with the increase of the studies, many researchers began to focus on the detection of anti-p53 antibody in liver cancer, but they have varying results.^[14,15]^ In the present study, we try to gather the whole studies on the detection of anti-p53 antibody by conducting a systematic review and meta-analysis to evaluate the diagnostic value of hepatocellular carcinoma.

## 2 Materials and methods

### 2.1 Literature search strategy

This meta-analysis was registered on PROSPERO (www.crd.york.ac.uk, ID: CRD42020171966). Two collectors (YC and BL) searched the studies on the serological detection of p53 antibody in human hepatocellular carcinoma in both Pubmed, Cochrane library, and Embase database. To ensure the integrity of searches, we mainly used 3 keywords, “hepatocellular carcinoma,” “liver cell cancer,” “anti-p53 antibody,” “blood or serum,” which limits were the time of January 1990 to December 2019 and non-English articles in both databases. Also, the relevant references in the articles were put into our study to increase the integrity of the included literatures. The online Supplemental Digital Content presents our search strategies (see Table S1, Supplemental Content, which illustrates the search strategies used).

### 2.2 Eligibility criteria

**Figure 1:**
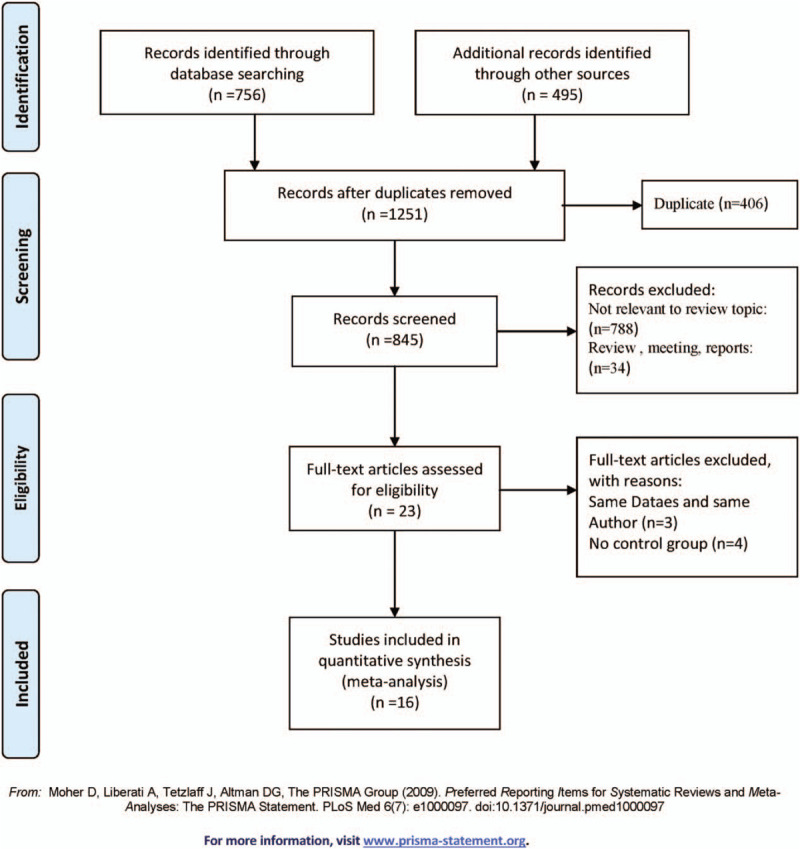
Flow chart of search history.

Figure 1 showed the search flow chart. All the records retrieved were completed independently by 2 different filters and disagreements were resolved by consensus. First of all, no important records, reviews, case studies, systematic reviews, studies of animal or cell lines, and other cancer literature studies are excluded. Then the inclusion criteria are as follows: The experimental group was diagnosed with HCC, and the clinical symptoms of the control group were different from that in the experimental group; anti-p53 antibody in serum or plasma of patients with liver cancer was detected by uncertain methods; the gold standard can be distinguished from the experimental group and the control group; TP (true positive), FP (false positive), FN (false negative), TN (true negative), which all can be calculated from the data provided, were diagnostic indicators of the anti-p53 antibody for HCC patients; The sample size of the whole experimental group should not be less than 20. The main exclusion criteria of the literature are as follows: one of the authors using the same experimental group or control group have been published; repetitive research; a great difference in the number of cases between the control group and the experimental group. The literature did not indicate the presence of the control group; the article does not provide sufficient information to make more Statistics with unreliable quality.

### 2.3 Evaluation of quality of literature

QUADAS (quality assessment for studies of diagnostic accuracy), recommended by Cochrane, which can be used in systematic reviews of diagnostic accuracy studies, was used to evaluate the methodological quality of the included studies by 2 independent reviewers. If there were differences in the processing, we would track it through the third as well. The QUADAS tool is based on a consensus reached by the 9 experts in the field of diagnosis including 14 evaluation items, respectively, the disease spectrum, gold standard, disease progression bias, confirmation bias, clinical evaluation bias, combined bias, case withdrawal, uncertain results, and detailed guidelines for scoring each of the items included in the tool.^[16]^ The evaluation of the tool has 3 results: “yes,” “no,” “not clear.” If the result of the evaluation is “yes,” the score of 1 article will be increased by 1 point, otherwise, it would get none.

### 2.4 Information extraction

Table 1 is completed independently by 2 data filters. YC and BL were respectively extracted data from the including 14 literatures with the standard form. Some of the differences were discussed with both 2. In the present study, the following features mainly include. Basic information: the first author, published years, country, region the collection time of the sample; the characteristics of the experimental group, the gold standard, sample group sex ratio, age, tumor classification, etiology; the disease spectrum in the control group; methodology evaluation of anti-p53: the test method of anti-p53 antibody, sample source, the value of cutoff, TP, FP, FN, TN. Not available was used to represent the contents if they were not mentioned in the original articles, and it should be noted that we have not contacted the author for more detailed information.

**Table 1:**
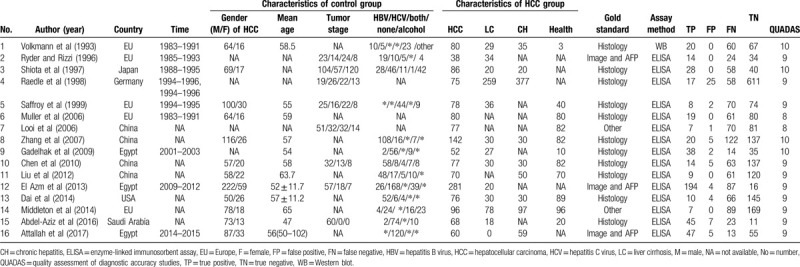
Characteristics of all included studies.

### 2.5 Statistical analyses

Through the 2 meta-data analysis methods in the diagnostic tests,^[17]^ Se and Sp are calculated and the forest map and the ROC curve of the subjects for each data including articles were drawn. The potential problem associated with Se and Sp was handled by adding 0.5 values to all cells of the diagnostic 2 × 2 table. Also, we are very interested in calculating the positive likelihood ratio (PLR), negative likelihood ratio (NLR), diagnostic odds ratio (DOR), and the 95% confidence interval (95% CI) of them. Q test can be used statistically whether there is the presence of heterogeneity between different results of these studies, and I^2^ can be used to reflect the effect size of the heterogeneity. I^2^ < 25%, showed no heterogeneity; I^2^ = 25% to 50%, indicating a moderate heterogeneity; I^2^ > 75%, indicating a high degree of heterogeneity.^[18]^ To investigate the source of heterogeneity among different experiments, meta-regression and subgroup meta-analysis were done by our group. All statistical procedures were performed in Stata15.1 (Corp. STATA, Station college, TX). The *P* values of the < .05 values of all the sets are statistically significant.

## 3 Results

### 3.1 Study characteristics and quality assessment

A total of 16 articles were included in this meta-analysis which was based on inclusion and exclusion criteria after completely searching. Table 1 shows the main information of the incoming articles, there are 1323 patients with HCC and the control group of 1896 patients with non-HCC, including liver cirrhosis, hepatitis, or normal healthy people in the meta-analysis.^[19–34]^ The gold standard of 12 studies was identified by histopathology as HCC and the other 4 studies^[21,26,31,33]^ were determined by clinical information, imaging, and AFP. Six were from Asia, 6 studies were from Europe, 3 studies were from Africa,^[21,24,33]^ and only 1 from the United States^[20]^ in all the 16 studies. In the aspect of etiology, the main causes of HCC were mainly found by virus infection (hepatitis B virus [HBV], hepatitis C virus [HCV]), alcoholic liver disease, and some other unknown factors. In particular, the HCV infection rate of the 2 studies in Africa that reached 50% was much higher than in other regions. Tumor grade here does not represent the same classification, which uses Barcelona Clinic Liver Cancer (6 articles were used Child-Pugh graded, and the other 3 articles were graded by other classifications). The control group was mainly composed of liver cirrhosis, hepatitis, and healthy people, but each of the personnel structures is not consistent, 10 contains easily confused liver disease and 3 had normal healthy people in the control group. The average score of the studies was 9 points, 4 articles were more than 9 points, and the other 2 were less than 9 points. The remaining articles were all of 9 points in the full 14 points score of the QUADAS tool. None of studies mentioned the statement of potential conflict of interests.

### 3.2 Diagnostic accuracy

**Figure 2:**
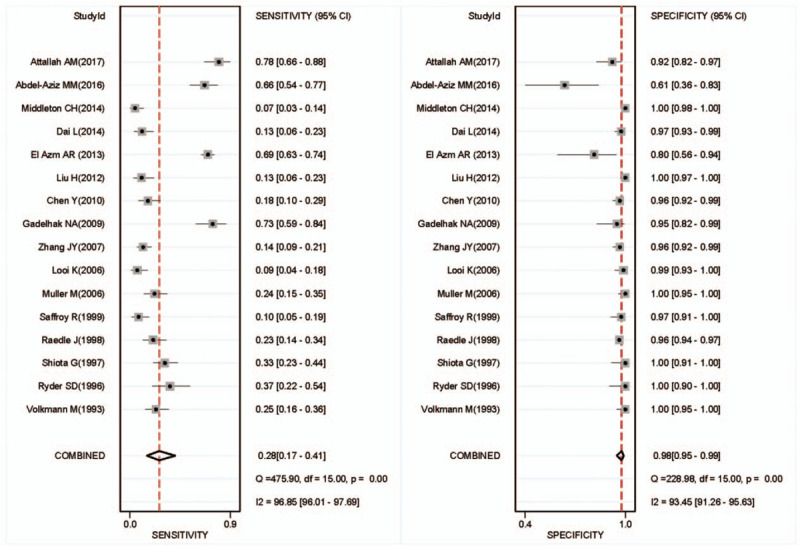
Forest plot of sensitivity and specificity of 16 individual studies for anti-p53 antibody in the diagnosis of HCC. The point estimates of sensitivity from each study are shown as solid circles. Error bars are 95% confidence intervals. HCC = hepatocellular carcinoma.

**Figure 3:**
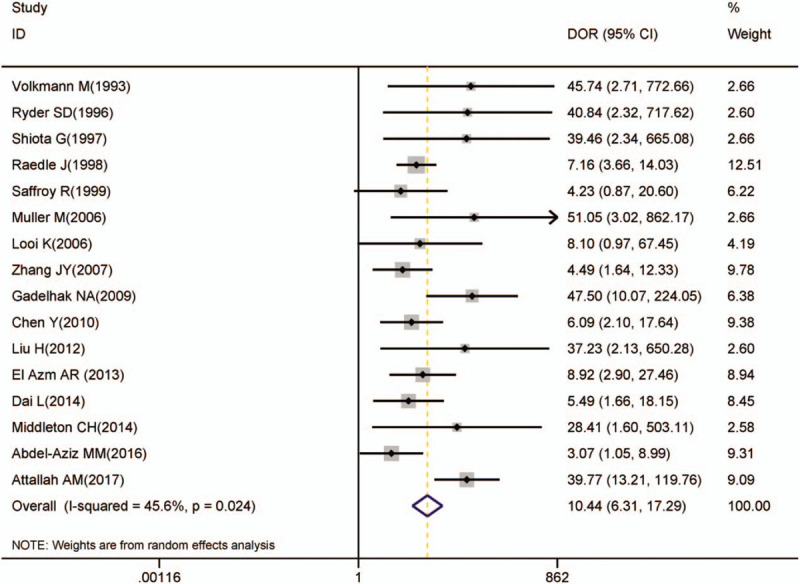
Forest plot of estimates of the diagnostic odds ratio for anti-p53 antibody in the diagnosis of HCC. HCC = hepatocellular carcinoma.

**Figure 4:**
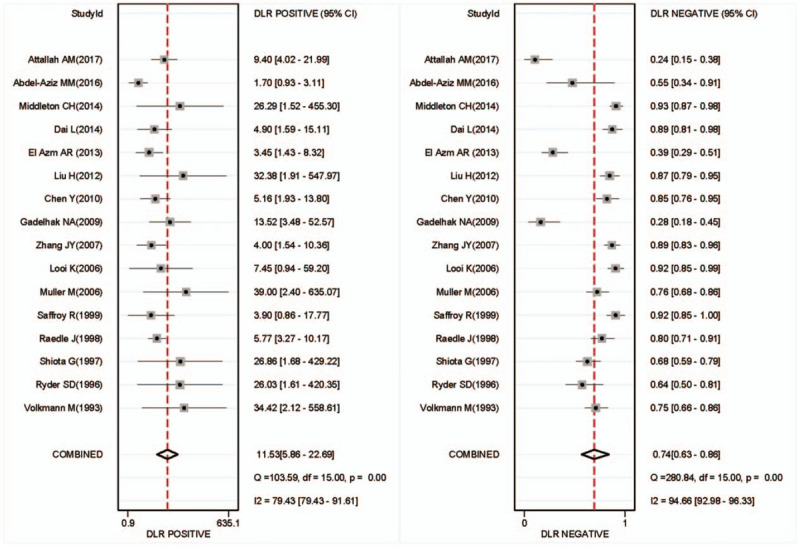
Forest plot of estimates of the positive likelihood ratio (PLR) and negative likelihood ratio (NLR) for anti-p53-antibody in the diagnosis of HCC. The point estimates of the positive likelihood ratio from each study are shown as solid circles. Error bars are 95% confidence intervals. HCC = hepatocellular carcinoma.

**Figure 5:**
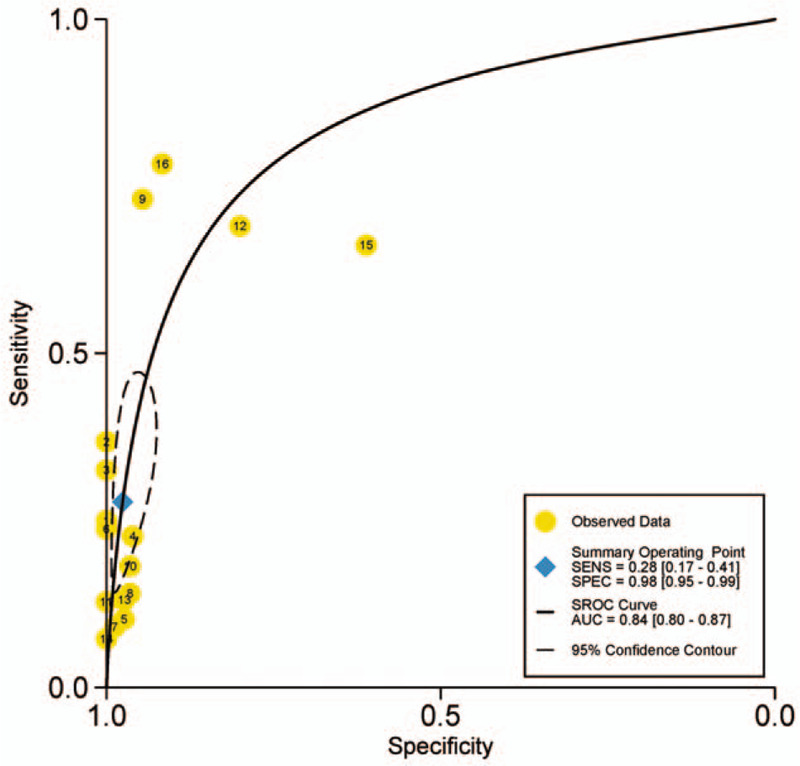
The sROC for anti-p53 antibody in the serum in the diagnosis of HCC. Each solid circle represents each study in the meta-analysis. HCC = hepatocellular carcinoma, SROC = summary receiver operating characteristic curves.

There was found the sensitivity of range 6% to 88% with a large difference and the pooled specificity was almost 95% after the summary of the 16 articles. The pooled sensitivity was 0.28(95% CI: 0.17–0.41, *P* < .001, I^2^ = 96.85%), and the pooled specificity was 0.98 (95% CI: 0.95–0.99, *P* < .001, I^2^ = 93.45%) (Fig. 2). Generally, it is considered that the sensitivity and specificity have a great relationship with the value of cutoff and may be unfavorable for evaluating the accuracy of the diagnosis experiment as the main index in the current study. Because the heterogeneity of the I^2^ and Q tests showed a little large heterogeneity, the random effect model was adopted in these articles. From the results of Figure 3, we can find that the pooled DOR was 10.44, 95% CI (6.31–17.29), I^2^ = 45.6% (*P* = .024), suggesting that including studies contained heterogeneity. Similarly, the pooled PLR was 11.53, 95% CI (5.86–22.69), I^2^ = 79.4%, *P* < .001, also showed a heterogeneity (see Fig. 4). The pooled NLR was 0.74 > 0.1, 95% CI (0.63–0.86), I^2^ = 90.3%, and also showed a large heterogeneity. In Figure 5, the AUC of symmetrical ROC was 0.840 > 0.7, indicating the medium value of the anti-p53 antibody in the diagnostic for HCC.

### 3.3 Possible sources of heterogeneity

Meta-regression and the subgroup analyses were used to explore the possible sources of heterogeneity, which include the region, proportion of HBV patients, proportion of HCV patients, number of HCC patients, the stage I% of the participants, and the style of negative control group composition. Meta-regression indicated that the above variables region, the proportion of HBV patients, and tumor stage I% may be the sources of heterogeneity for anti-p53antibody in HCC serum (*P* < .001). The results of the subgroup analysis were shown in Table 2, which contained region, HCV, HBV, stage I%, number of HCC patients, and control style. It should be noted that the subgroup of African studies, tumor stage I < 50%, HBV patients ≥50% had a low degree of heterogeneity. Besides this, subgroup performed in HCV patients proportion ≥50% has a higher sensitivity (0.58, 95% CI: 0.32–0.81), despite the heterogeneity.

**Table 2:**
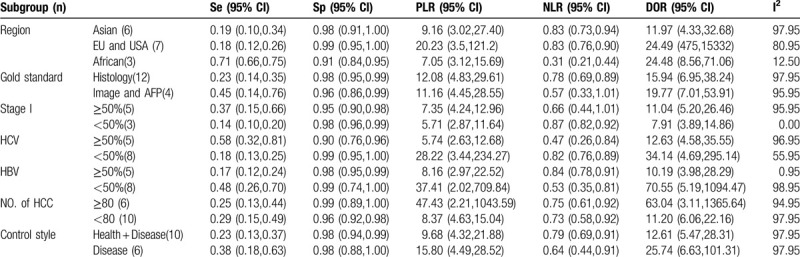
Subgroup analysis of the available information of anti-p53 antibody in these articles for HCC.

### 3.4 Publication bias and sensitivity analysis

**Figure 6:**
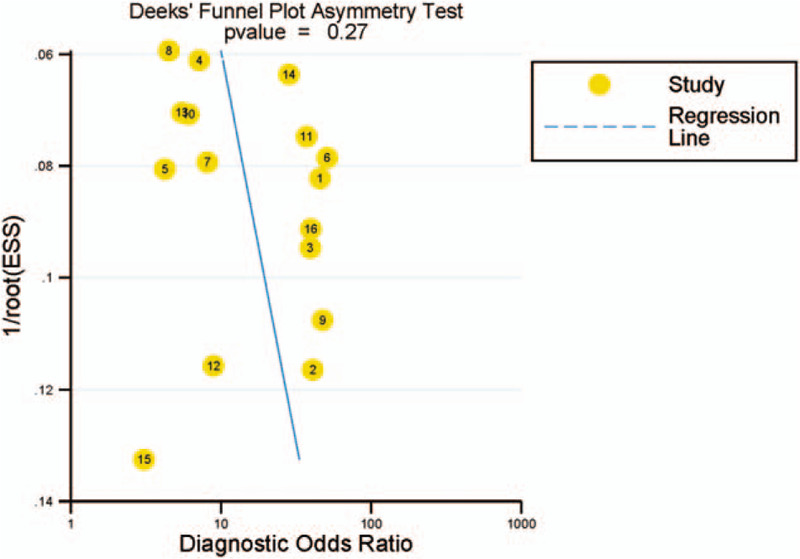
Publication bias of 16 articles with the diagnostic odds ratio.

Overall, it may appear to be a certain degree of publication bias in different aspects of these studies. The most obvious is that the sensitivity of Asian and European studies showed lower than that in the African studies. However, Deeks’ funnel plots for publication bias also showed symmetry in Figure 6, indicating that these studies had no publication bias (*P* = .270).

Sensitivity analysis was conducted in terms of statistical analysis methods, sample size, QUADAS, and so on. However, the results produced no obvious changes by using a random effect model to replace the fix effect model.

## 4 Discussion

In the present study, 16 studies on the detection of serum anti-p53 antibody were collected for HCC by our group. According to the findings, we could regard it as the first meta-analysis of anti-p53 for HCC. The detection of anti-p53 antibody in the diagnosis has a moderate value for hepatocellular carcinoma (AUC = 0.840) in the summary receiver operating characteristic curves (sROC), suggests that the anti-p53 antibody may become an effective marker for the diagnosis of HCC. However, the low sensitivity is not perfect and would limit the clinical application.

Usually, the pathological examination is the gold standard for HCC. However, liver cancer patients with the same clinic pathologic features often display different results, which suggest that there are several complex molecules and cells involved in the development of hepatocellular carcinoma. It's still lack of effective diagnostic markers for the early diagnosis. Although the current tumor markers, such as AFP, AFP-L3, and DCP, play an important role in the diagnosis of HCC, they are mostly used in conjunction due to lack of specificity.^[35–38]^ Along with the antigens of tumor cells were easily changing, anti-tumor-associated antigens (TAA) may be detected before symptoms appear in 5 years, demonstrating that the body could be an immune reaction to that abnormal auto-antigen early. In the past few years, an increasing number of studies have focused on the immune response of the body to the tumor, and the detection of autoantibodies to tumor-associated antigens has been carried out in which the highest focused point is the anti-p53 antibody in various cancers.^[15]^ As a tumor suppressor gene, the mutation of the p53 gene is found not only in HCC but also in many other cancers. The anti-p53 antibody is not specific for HCC, the other as esophageal carcinoma,^[39]^ gastric carcinoma,^[40]^ colorectal carcinoma,^[41]^ and other cancer patients can be detected as well. P53 gene mutation and antibody can be coexisting, but not completely consistent in different cancers, suggesting that the anti-p53 antibody may be one of the diagnostic indicators of some cancers.

As a whole, the Sp of anti-p53 antibody for HCC is generally coming to 98% (*P* < .001), but Se had a wide range of 6% to 88% in each study (*P* < .001). The problem of heterogeneity between NLRs is a key point to our Meta-Analysis. A gold standard is one of the important steps of the experimental diagnosis. The 4 included articles of them did not fully use pathological to diagnosis which would inevitably affect the results of the experiment. Enzyme-linked immunosorbent assay (ELISA) is one of the most convenient and simple tests which is the only way to detect serum anti-p53 antibodies in these included studies excluding the western bolt. Another reason for heterogeneity is the difference of population selection in the control group. There were 2 studies only using the normal healthy samples as the control group significantly increased the ability of anti-p53 antibody in the diagnosis of HCC in different liver diseases.^[26,28]^ In addition, it can be seen in the study of tumor grade (only 7 studies) that the diagnostic efficacy of anti-p53 in patients with primary hepatocellular carcinoma was slightly higher than that in late stage, but the difference between them was not obvious. Other factors such as age, the sex ratio in the analysis of our meta-regression also express a certain significance. In our opinion, these factors mentioned above are the causes of heterogeneity. Although we try our best to find the heterogeneity, it cannot come to the completely reliable conclusion because of the lack of information. In fact, HCC is extremely heterogeneous cancer, so it cannot thoroughly eliminate the heterogeneity of Se, Sp, and NLRs in all studies.

In particular, the Se of anti-p53 antibodies in several African studies are much higher than the Asian studies, but their specificity was significantly lower than in other regions. Some studies show that the HCV core protein gene can induce the mutation of the p53 gene and HCV Core protein can be involved in the regulation of p53 gene expression, which leads to the generation of abnormal p53 protein in human body.^[42]^ We found that the HCV infection rate in Egyptian patients with liver disease leads to a relatively high positive rate, which is an important reason for the impact of heterogeneity.^[21,24,43,44]^ Simultaneously, HBV was one of the most high-risk factors of HCC which infected about 90% HCC patients in Asia especially China. Some researchers suggested that HBV X protein through inhibiting p53 gene expression^[45]^ may be caused by the lower rate detection of anti-p53 antibody in the serum of HCC.

After analyzing the heterogeneity of the study, it cannot ignore the value of PLR, DOR (Fig. 4), and sROC curve (Fig. 5) of the anti-p53 antibody for diagnosis for HCC. The likelihood ratio is an indicator that needs to be concerned which can deeply reflect the validity of a diagnostic test. If PLR or DOR is greater than 10, or NLR is less than 0.1, it is suggested that the method is a reliable diagnostic test. The detection of the anti-p53 antibody in the diagnosis has a moderate value for HCC (AUC = 0.840) in the sROC. However, the pooled DOR is 10.44, which is greater than 10 with a small heterogeneity, suggesting that the anti-p53 antibody may become an effective marker for the diagnosis of HCC.

Although previous studies have not indicated that the anti-p53 antibody can serve as a perfect diagnostic marker for HCC, it has brought a lot of inspiration to the later researches. In the future, ELISA is still an important serological method for detecting antibodies for HCC, but it will be more convenient and faster than before. Since the combination diagnosis has been an important method for the diagnosis of disease. The diagnostic effectiveness of anti-p53 antibody combined with AFP has been verified. Fortunately, these markers of HCC could maintain high Sp, which improves the Se of the diagnostic test, are increasing the interest of many researchers at the same time. Especially, the anti-p53 antibody can be used to observe the prognosis of HCC patients, which provides a broader perspective for the study of anti-p53 antibody. At present, there are some studies that have begun to research anti-p53 antibody in the prognosis,^[46–49]^ but too rare and they cannot fully explain the effect of anti-p53 antibody.

## 5 Conclusion

Current evidence suggests that the anti-p53 antibody may be a potential indicator of potential value in the diagnosis of hepatocellular carcinoma, and has a certain clinical value. Despite the high specificity of the anti-p53 antibody for HCC, the low sensitivity is not perfect and would limit the clinical application. The anti-p53 antibody would help rule out HCC but not help rule in HCC for early diagnosis. Thus, a combination of panels of TAAs to detect multiple different and specific antibodies is the current goal to increase their diagnostic potential for HCC.

## Author contributions

YC and HL contributed to the study design. YC and BQL generated the literature strategy and filtered through the identified studies. HYN and SHX evaluated study quality. Study results were entered in duplicate by HL, SHX, and YC. All authors read and approved the final manuscript.

## Supplementary Material

Supplemental Digital Content
