# Supplementary material for: Value of anti-p53 antibody as a biomarker for hepatocellular carcinoma: Evidence from a meta-analysis
Source: Medicine (Baltimore). 2020 Aug 21;99(34):e21887. doi: 10.1097/MD.0000000000021887 (PMC7447394; doi:10.1097/MD.0000000000021887)
Supplement: Supplemental Digital Content [file medi-99-e21887-s001.docx]

**Supplementary Table1. The search strategies in database.**

| **Database** | **Search terms** |
| --- | --- |
| Pubmed | 1#Carcinoma, Hepatocellular [Mesh] OR Hepatocellular Carcinomas OR Liver Cell Carcinoma, Adult OR Liver Cancer, Adult OR Adult Liver Cancer OR Adult Liver Cancers OR Cancer, Adult Liver OR Liver Cancers, Adult OR Liver Cell Carcinoma OR Carcinoma, Liver Cell OR Cell Carcinoma, Liver OR Cell Carcinomas, Liver OR Liver Cell Carcinomas OR Hepatocellular Carcinoma OR Hepatoma OR Hepatomas |
|  | 2#p53 autoantibody OR tumor-associated antigens OR p53 antibody OR p53 OR p53 autoantibodies OR p53 AAb OR p53 Ab OR TP53 Protein |
|  | 3# Serum OR blood |
|  | 1#&2#&3# |
| Embase | protein p53'/exp OR 'tp53' OR 'p53' AND ('antibody'/exp OR 'autoantibody'/exp OR 'autoantibodies' OR 'antibodies') AND ('liver cell carcinoma'/exp OR 'hepatocellular carcinoma' OR 'liver cancer'/exp OR 'hepatomas' OR 'adult liver cancer') AND (‘Serum’ OR ‘blood’) |
| Limits | Publication date:1990-2019 Language : English articles Source：only human |
